# New insights into retinal circuits through EM connectomics: what we have learnt and what remains to be learned

**DOI:** 10.3389/fopht.2023.1168548

**Published:** 2023-04-20

**Authors:** Abhilash Sawant, Aindrila Saha, Jacob Khoussine, Raunak Sinha, Mrinalini Hoon

**Affiliations:** ^1^ Department of Ophthalmology and Visual Sciences, University of Wisconsin-Madison, Madison, WI, United States; ^2^ Department of Neuroscience, University of Wisconsin-Madison, Madison, WI, United States; ^3^ Cellular and Molecular Biology Program, University of Wisconsin-Madison, Madison, WI, United States; ^4^ McPherson Eye Research Institute, University of Wisconsin-Madison, Madison, WI, United States; ^5^ Medical Scientist Training Program, University of Wisconsin-Madison, Madison, WI, United States

**Keywords:** retina, electron microscopy, circuit, synapse, reconstruction

## Abstract

The retinal neural circuit is intricately wired for efficient processing of visual signals. This is well-supported by the specialized connections between retinal neurons at both the functional and ultrastructural levels. Through 3D electron microscopic (EM) reconstructions of retinal neurons and circuits we have learnt much about the specificities of connections within the retinal layers including new insights into how retinal neurons establish connections and perform sophisticated visual computations. This mini-review will summarize the retinal circuitry and provide details about the novel insights EM connectomics has brought into our understanding of the retinal circuitry. We will also discuss unresolved questions about the retinal circuitry that can be addressed by EM connectomics in the future.

## Introduction

Connectomics is a branch of neuroscience focused on the study of neural connections. High-throughput reconstructions of neural circuits through electron microscopy (EM) have enabled considerable advances in mapping new connections across neural circuits and also unveiled new features about existing neural connections. The use of 3D EM to unravel neural connections has advanced our understanding of the neural composition and connectivity in the mammalian retina. The retina is a specialized tissue and the neuronal populations in this tissue are tasked with the responsibility of converting visual features into electrical signatures that are in turn transmitted to higher brain areas. Intricacies of the retinal circuitry have been revealed with EM connectomics and this mini-review will highlight some of the advances in this field. We will detail the basic design principles of the mammalian retina and highlight connectomic studies from the mouse and primate (macaque and human) retina that have provided new insights into the retinal circuitry. Common 3D EM techniques for circuit reconstructions are: (i) serial transmission EM studies that rely on serial sectioning followed by imaging with a transmission electron microscope or TEM, (ii) serial block face scanning EM that rely on simultaneous sectioning and imaging of a block of tissue with a scanning electron microscope or SEM outfitted with an ultramicrotome and, (iii) automated tape collecting ultramicrotomy followed by SEM imaging (1).

**Figure 1:**
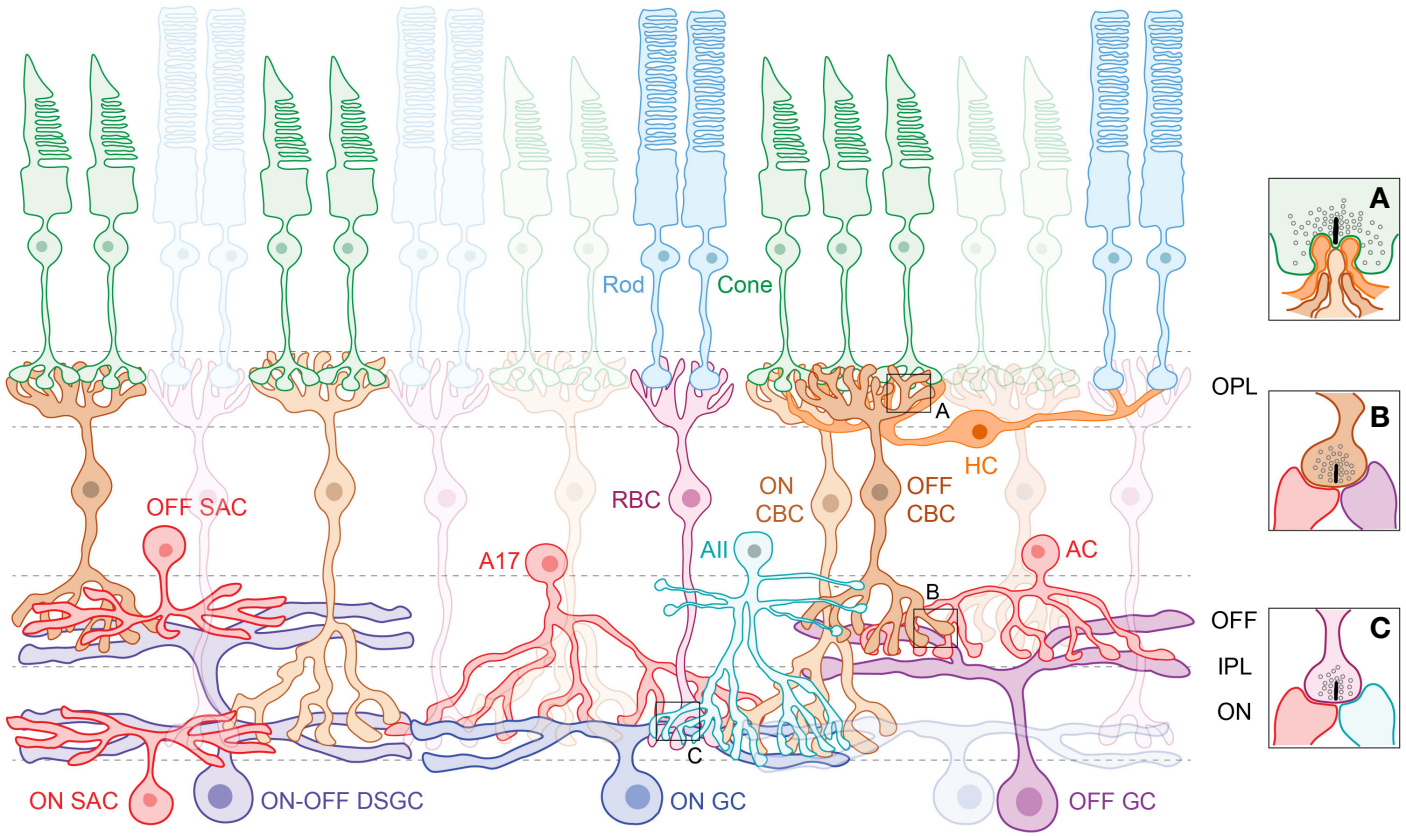
The retinal schematic highlights the cellular diversity and the need to trace physical connections between neurons and circuits. Photoreceptors in the outer retina, the rods and cones, synapse with respective rod (RBC) and cone bipolar cells (CBC) and form ribbon synaptic ‘triads’ with horizontal cell (HC) interneurons **(A)** in the outer plexiform layer (OPL). Amacrine cell (AC) interneurons modulate information flow in the inner plexiform layer (IPL), where the RBCs and CBCs relay signals to retinal ganglion cells (GCs). Note that CBCs synapse with both ACs and GCs **(B)** at ribbon synaptic ‘dyads’, whereas RBCs synapse exclusively with ACs **(C)**. Processes of retinal neurons are segregated into distinct ON and OFF sublaminae in the IPL – ON cells that depolarize to light increments laminate deeper in the IPL, whereas OFF cells that depolarize to light decrements laminate superficially. Subtypes of ACs serve specific visual functions, such as the AII, which conducts rod-driven signals to retinal output pathways, the A17 that provides feedback inhibition onto the RBC, and the starburst amacrine cells (SACs) that are integral to the direction-selective (DS) circuit (*Left*). There are a broad variety of ON GCs, OFF GCs and ON-OFF GCs that stratify across the IPL. GCs such as the ON-OFF DSGC integrate input from ON and OFF bipolar cells and ON and OFF amacrine cells to perform visual computations.

*The retinal circuit:* The retina is a multi-layered organized neural tissue at the back of the eye (**Figure 1**) (2, 3). Conversion of photons into electrical signals begins at the photoreceptors that are of two main types – rods that are operational at dim-light setting and cones that support color vision and are operational at bright light conditions. Each photoreceptor type is connected to dedicated second order retinal neurons - bipolar cells (BCs) and horizontal cells (HCs). BCs ferry photoreceptor signals from the outer retina to amacrine cell (AC) interneurons and ganglion cell (GC) output neurons in the inner retina, whereas HCs modulate processing at the outer retina. There are several subtypes of BCs, amacrine interneurons, and GCs that support the processing of distinct visual features such as coding increments (ON) vs decrements (OFF) of light, directional information or processing of dim-light (night vision) signals (4). There are also specialized ganglion cells that contain the visual pigment melanopsin (5) and are intrinsically photosensitive (6). These cells are responsible for non-image forming function such as circadian photoentrainment. Overall, retinal circuits exhibit diverse patterns of synaptic connectivity and organization, which are optimized to their functional needs.

## Understanding the retinal circuitry through connectomics

The use of EM connectomics has revealed new cell types that constitute the retinal circuit. The first 3D volume EM reconstructions of retinal neurons provided a catalog of cell types that are dedicated to diverse visual pathways in the inner retina (7–9). This generated a map of inner retinal neurons which has since served as a framework for identifying and classifying retinal neurons. Summarized below and in **Table 1** are some of the advances offered by EM connectomics for increasing our understanding of retinal circuits.

**Table 1:** Table summarizing some of the advances in retinal circuitry through serial EM reconstructions.

Circuits	Species	Findings via serial EM	Reference
Cone Pathways and Outer Retina	Mouse	*Connectivity of rod photoreceptors with OFF cone bipolar cells.*	(10)
		*Photoreceptor to bipolar cell contact profiles in the outer retina.*	(11)
		*Classification of cone BC types and connectivity with the AII AC.*	(12)
		*Assessment of rod/cone gap-junctional coupling in the outer retina.*	(13)
		*Classification of synaptic ultrastructure and connectivity motifs of axon terminals across cone BC types.*	(14)
	Primate	*Bipolar cell input to the midget GCs in the parafoveal region of the human retina.*	(15)
		*Characterization of synaptic connectivity in the midget circuitry of the primate fovea.*	(16)
		*Identification of the S-Cone OFF midget private line connection in the foveal macaque retina.*	(17)
		*Connectivity of rod photoreceptors with an OFF cone bipolar cell-type.*	(10)
		*Connectivity of OFF BC types in the inner and outer macaque retina.*	(18)
		*Classification of cone BC types and their synaptic connectivity in the perifoveal macaque retina.*	(19)
		*S-Cone OFF midget connectivity in the parafoveal macaque retina.*	(20)
		*Development of the foveal midget circuitry in the human retina.*	(21)
Night-Vision Circuit	Mouse	*Connectivity and synaptic ultrastructure of the AII AC.*	(12)
		*Connectivity between the AII AC and the OFF cone BCs.*	(22)
		*Development of partner-specific synaptic connectivity of the AII AC.*	(23)
		*Identification of the primary inhibitory input to the AII cell: the nNOS-1 AC.*	(24)
		*Role of presynaptic inhibition in maintaining ultrastructure of rod BC terminal synapses.*	(25, 26)
		*Preferential connectivity of the A17 AC across rod and cone pathways.*	(27)
		*Identification of dendro-somatic inhibitory input to the OFF-sustained alpha GCs from the AII ACs.*	(28)
	Primate	*Assessment of rod BC connectivity in the inner and outer retina.*	(29)
Direction Selectivity Circuit	Mouse	*Asymmetric connectivity pattern of SAC inhibitory input to the DSGCs.*	(7)
		*Specificity of Off bipolar cell contacts with SACs.*	(30)
		*Specificity of ON bipolar cell contacts with SACs.*	(31)
		*Identification of novel widefield amacrine cells (WACs) that provide input to cone BCs in the DSGC circuit.*	(32)
		*Distribution of SAC synaptic input and output profiles.*	(33)
		*Identification of novel synaptic motifs involving WACs in the ON and OFF layers that underlie ON-OFF DSGC contextual sensitivity.*	(34)
		*Asymmetric distribution of cone BC input to the ON DSGCs.*	(35)
		*Identification of a novel synaptic motif involving WACs and SACs that provides input to type 2 and type 7 cone BC terminals.*	(36)
		*Identification of three narrowfield ACs that provide synaptic input to the SACs.*	(37)
	Primate	*Morphological characterization of an ON-DSGC in the primate retina: the recursive monostratified (rmGC) circuit.*	(38)
		*Morphological characterization of an ON-OFF DSGC in the primate retina: the small bistratified GC circuit.*	(39)
Melanopsin Pathway	Mouse	*Identification of bipolar cell-types that provide input to an ipGC subtype (M5).*	(40)
		*Axonal arborization and ultrastructure of ipGC synaptic inputs across higher brain areas.*	(41)
	Primate	*Identification of a novel S-cone ON AC that provides input to the M1 ipGCs.*	(42)
		*Identification of a novel synaptic target of the S-ON BC: the M2 ipGC.*	(43)
		*Identification of cell-types that provide excitatory and inhibitory input to the displaced M1 ipGCs.*	(44)
Other Signaling Pathways	Mouse	*Identification of the Type X Cone BC (XBC) and sub-classification of type 5 BCs.*	(8)
		*Characterization of a novel monopolar glutamatergic interneuron (GluMI).*	(45)
		*Characterization of two novel retinal ganglion cell-types and identification of a novel* *BC type.*	(46)
		*Identification of an ON-laminating widefield AC that provides input to the ON-Alpha GC* **via* dual release sites.*	(47)
		*Characterization of a novel Müller glia-coupled AC (MAC).*	(48)
		*Identification of vertically-oriented AC processes that contact the type 5A cone BC* *axon terminals.*	(49)
	Primate	*Assessment of synaptic input to the ON Parasol GCs.*	(50, 51)
		*Identification of 2 widefield ACs that provide input to the ON Parasol GCs: the wiry type 2 and semilunar type 2 ACs.*	(52)
		*Identification of AC types that provide dominant synaptic input to the broad thorny GC* *in the central macaque retina.*	(53)

### Cone pathways and outer retina

Rod and cone photoreceptors form synaptic ‘triad’ connections with BC and HC processes in the outer retina (**Figure 1**). Rods and cones are also connected via gap-junctions, which enable electrical communication (2). EM datasets have enabled study of the gap-junctional coupling in the outer retina revealing that in mouse retina the majority of gap-junctions are heterologous (i.e., rod-cone) and that a single cone photoreceptor on average makes ~50 gap-junctions vs ~2-3 made by rod photoreceptors (13). Another study utilized the same dataset to map the connectivity between photoreceptors and downstream BCs and suggested novel instances of apposition between photoreceptors and BC types (11). This study also found that some cone BC types contacted fewer cones than expected and one ON cone BC type made atypical contacts with cone photoreceptors (11). Another serial EM study in the mouse retina confirmed the classification of 15 BC types and determined their connectivity with a specific downstream AC called the AII AC (12). They showed that the AII AC contacts most (5 out of 6) OFF BC types via chemical synapses and 7 out of 8 ON BC types via gap-junctional coupling, with the Type 2 OFF (69 percent of synapses) and Type 6 ON BCs (46 percent of gap-junction area) being the major partners. The output profiles of cone BC types in the mouse retina have been recently detailed through serial EM reconstructions. Notably, the cone BCs provide ribbon synaptic input to ACs and GCs via six different structural motifs, with each cone BC preferring a specific subset of motifs as their preferred mode of output (14). Together, our understanding of the ultrastructural ‘units’ of retinal connectivity have been enhanced through EM connectomics.

In the primate retina, serial EM studies in the foveal region, which exhibits the greatest cone photoreceptor density and is responsible for the highest acuity vision (54), have revealed the connectivity pathway for short-wavelength (S) cone photoreceptors and the developmental timeline for wiring of foveal circuits. Terminals of S-cone photoreceptors are smaller and contain more synaptic ribbons than neighboring cone terminals (17) and these terminals establish a 1:1 connectivity with OFF midget BCs and OFF midget GCs excluding contact with ON midget cells (17, 20). Early serial TEM studies identified the unique synaptic configuration of the numerically dominant neural circuit in the macaque fovea called the ‘midget’ pathway (16). This pathway exhibits a 1:1 synaptic connectivity between cone photoreceptors and downstream midget BC and midget GCs enabling a ‘private-line’ of communication from cones to output neurons (15, 16). A recent study by Zhang and colleagues determined the developmental timeline of the foveal midget circuitry in human retina (21). They reconstructed the morphological and synaptic features of this circuit in the developing human fovea and found that by fetal week 14 the cone to midget BC connectivity was comparable to adult and by fetal week 21 the cone-BC-GC private line was established. This work further revealed that the OFF midget pathway is established prior to the ON pathway. Serial EM studies in primate retina have also identified the connectivity and arrangement patterns of synaptic inputs onto several ON and OFF BC types (10, 18, 19).

### The night vision circuit

The night vision circuit commences with rod photoreceptors that connect to rod BCs in the outer retina. Rod BCs in turn provide output to two AC types – AII and A17 ACs in the inner retina. Rod signals from AII AC are routed to output GCs via cone BC terminals whereas the A17 provides feedback inhibition onto rod BC terminals. In the macaque retina, Grunert and Martin used serial EM to illustrate the traditional rod BC to AII synapse in the inner retina (29). They reported instances of rod BC excitatory inputs to AII AC at ribbon synapses with adjacent A17 AC forming reciprocal inhibitory synaptic connections onto the rod BC terminal. Through serial EM studies, new principles about the organization of the rod BC output ribbon synapses have been uncovered in the mouse retina. Specifically, rod BC terminals with impaired synaptic inhibition fail to form stereotypic output connections and instead engage in erroneous synaptic contacts with AII and A17 cells (25, 26). EM reconstructions of the developing AII circuit have shown that the AII ACs preferentially increase chemical synaptic connections with OFF BCs, eliminate connections with widefield ACs and maintain their original synaptic output onto GCs during development (23). Connectomic studies on the AII have also detailed the preference of the AII AC to contact specific OFF BC and GC types (12, 22, 28). Serial EM studies have added further details about the mouse night vision circuit and shown that the AII AC output is primarily regulated by inhibition through a multi-stratified nitric oxide synthesizing (nNOS-1) AC (24). Serial EM studies have also elaborated on the connectivity profiles of the A17 AC demonstrating that the A17 AC has an intrinsic bias towards contacting rod BCs and providing feedback inhibition. However, this preference shifts to the cone BCs when rod BCs are ablated from the circuit, indicating a hierarchical selection process that dictates connectivity of this AC (27).

### The direction selectivity circuit

The ganglion cells encoding motion direction in the retina, the direction-selective GC (DSGCs), were discovered decades ago (55). Subsequent efforts to understand the mechanisms underlying computation in this circuit elucidated key components of the circuit including the role of GABAergic inhibition from the starburst ACs (SACs) in suppressing the response of the DSGC when stimuli moved in an unpreferred or ‘null’ direction (56). Ultrastructural information about the synaptic connectivity and morphological arrangement of SACs with DSGCs through EM connectomics has provided new insights into the organization of this circuit. 3D EM reconstructions, after identification of DSGCs via calcium imaging, enabled visualization of the mouse DS circuitry, informing us about the asymmetric connectivity between SAC dendrites and DSGCs along the null direction (7). Further studies on this EM dataset assessed contacts between cone BCs and SACs including lamination depth and contact areas between cone BCs and OFF and ON SACs (30, 31). A subsequent study with a new EM volume determined that the Type 1 and Type 3a cone BCs provide synaptic input to the OFF SACs, and the Type 7 and Type 5 cone BCs provide input to the ON SACs at the proximal and distal dendritic ends, respectively (33). This dataset has been used further for addressing additional questions about the DS circuitry. The spatial extent of ON cone BCs was mapped onto ON DS cells, demonstrating a striking asymmetry of BC input along the directional axis with distinct Type 5 ON cone BC subtypes tuned to the preferred and/or null-side (35). A subsequent study determined that the Type 2 OFF and Type 7 ON cone BC axon terminals are directionally tuned owing to complex inhibitory synaptic motifs comprising SACs and widefield ACs (WACs) (36). Serial EM studies have unveiled additional details about the connectivity of WACs in the inner retinal DS circuit (32, 34) and have also revealed inhibitory input onto ON SACs from a class of narrowfield ACs (NACs) (37). Together, these studies have used EM connectomics to expand our understanding of the DS circuit in the mouse retina.

In primate retina, serial EM has enabled identification of DSGC types. Whereas SACs had been identified for the primate retina, the other components of the DS circuit had remained unknown until recent connectomic studies. Patterson and colleagues combined retrograde tracer injections into the superior colliculus and serial EM to identify recursive monostratified GCs (rmGC) as the primary ON DSGC candidate in the primate retina (38). The dendritic morphology, stratification and stereotypic SAC synaptic input onto rmGCs matched what has been reported for DSGCs in other vertebrate species. This study also detailed the pattern of SAC and BC input onto rmGCs in primate retina. A parallel study combining functional recordings with serial EM reconstructions revealed further components of the DS circuit in primate retina, including an ON-OFF DSGC and a bistratified polyaxonal AC, which were direction selective in addition to the SAC (39). This study also mapped the organization of BC input onto SAC underlying the basis of motion computation in the primate retina.

### The melanopsin circuit

Intrinsically photosensitive ganglion cells (ipGCs) form the basis of non-image forming vision (57). Recent connectomic studies have elucidated the identity of the BC types that provide input to ipGC types (M1-M6) in the mouse retina and also revealed the pattern by which ipGC axons contact their synaptic partners in higher brain regions. These studies have shown that the M5 ipGCs receive input from axon terminals of Type 6-9 BCs (40). Another study performed correlative light-electron microscopy to determine the ultrastructural features of contacts made by the ipGC axons at higher brain areas and found that the ipGC axons form synaptic boutons of different volumes and contact different numbers of postsynaptic partners at different brain regions via distinct connectivity motifs (41). These features of connectivity were discernable through serial EM reconstructions.

Multiple subtypes of ipGCs have been defined in the macaque primate retina, but the class of ipGCs with somas ‘displaced’ to the inner nuclear layer have evaded substantial investigation. Bordt and colleagues used serial EM to determine the synaptic inputs to the displaced ipGCs in central macaque retina and found that the majority of the synaptic inputs to these ipGCs came from ACs. This study also characterized the BC input to the ipGCs (44). Patterson and colleagues used serial EM to determine how short wavelength spectral information could influence circadian biology. This group reconstructed S-cone ON BCs via serial EM in the central macaque retina and found that these cells provide input to M2 ipGCs (43). Similar studies also revealed the circuitry providing input to M1 ipGCs in the primate retina (42). Together, serial EM reconstructions have expanded our knowledge about the retinal synapses and circuits that modulate circadian rhythms.

### Other signaling pathways

Serial EM reconstructions have revealed the presence and connectivity of new retinal cell types. The first study mapping the inner retinal connectome generated a high-resolution structural map of ~950 neurons in the mouse retina (8). This study found a new retinal BC type and revised the classification of cone BC types. A novel OFF cone bipolar cell type (Type 0) was uncovered through connectomic studies in the mouse retina showing that it provides input to R-cells, which are a type of bistratified GC (46). Serial EM studies have also revealed unique profiles of AC input onto BC terminals (49). A serial EM study reconstructing the inhibitory AC-driven input onto the ON alpha GC in the mouse retina found a new ON-laminating widefield AC (ONWAC) that provides input to the GC at specialized synapses utilizing dual vesicle release sites (47). Thus, serial EM can reveal the identity of new circuit components as well as detail their ultrastructural synaptic features. Serial EM reconstructions have also mapped the sites of synaptic input and output across unique retinal cell types such as the glutamatergic monopolar interneuron (GluMI) and the Muller-glia coupled Amacrine Cell (MAC) in the mouse retina (45, 48). This has enabled identification of the circuit partners of these cell types thereby revealing their potential roles in visual information processing.

In the primate retina, serial EM reconstructions have identified two types of widefield amacrine cells (WACs – the Type 2 semilunar and the Wiry Type 2 cell) that provide the majority of the inhibitory input to the ON parasol GCs (52). The BC input onto ON parasols has also been determined by serial EM studies (19, 50, 51). Together these studies have revealed the connectivity motifs between these cell types in the primate retina, highlighting new insights into the signaling mechanisms supporting visual computations in this circuit. Bordt and colleagues similarly used serial EM to classify the diverse AC input to another primate GC type – the broad thorny GC (53). Serial EM reconstructions in the macaque peripheral retina also identified a S-cone specific AC that exclusively receives excitatory glutamatergic input from S-cone ON BCs (42). This work also showed that this novel AC provides inhibitory input to the M1 ipGC with high specificity, which could underlie circadian entrainment in primates. Since it is not obvious that there should be a color vision circuit driving non-image forming visual behaviors, the ability to trace the physical connections between individual neurons underlying the circuits contributes important information that might otherwise be overlooked. Together, EM connectomic approaches are powerful tools to map retinal circuits, identify connectivity features, and unravel the ultrastructural correlates supporting retinal function and visual behavior.

## Discussion

There remain open questions about the retinal circuitry and connectivity, including synaptic organization, that can be addressed through EM connectomics in the future. For example, details about the connectivity motifs used by primate retinal neurons across retinal locations remain to be thoroughly characterized, and serial EM approaches are key to support these studies. Future connectomic studies using serial EM would be essential to tease apart differences in species-specific wiring of conserved retinal circuits as recently demonstrated for the DSGC circuitry (33). Furthermore, serial EM techniques can advance our understanding of how retinal diseases impact the ultrastructural features of connectivity amongst retinal neurons that could be the basis of the functional aberrancies observed in these conditions. Given the rich diversity of retinal neurons, new retinal cell types and circuits remain to be discovered through reconstructions of serial EM datasets that include correlative light and electron microscopy. Connectomics can also reveal new insights into the contact partners and contact type of GC projections to higher brain areas providing key mechanistic insights into the assembly of the visual pathway.

## Author contributions

All authors listed have made a substantial, direct, and intellectual contribution to the work and approved it for publication.
